# Investigator Responsibilities in Clinical Research

**DOI:** 10.31486/toj.19.0085

**Published:** 2020

**Authors:** Amy K. Feehan, Julia Garcia-Diaz

**Affiliations:** ^1^ Department of Infectious Disease, Ochsner Clinic Foundation, New Orleans, LA; ^2^ The University of Queensland Faculty of Medicine, Ochsner Clinical School, New Orleans, LA

**Keywords:** *Clinical trial*, *disclosure*, *drugs–investigational*, *government regulation*, *investigational new drug application*, *research personnel*

## Abstract

**Background:** Clinical trials are an integral part of translating new basic science research into therapeutics. It is crucial for those who run clinical trials to realize the gravity of their responsibilities as principal investigators.

**Methods:** This review focuses on the relevant investigator responsibilities under the Code of Federal Regulations Title 21, the contents of Form 1572, FDA inspections, and methods to improve compliance.

**Results:** While responsibility for day-to-day study activities can be delegated to outside entities and to study staff, a clinical principal investigator who has signed US Food and Drug Administration (FDA) Form 1572 is held responsible for noncompliance and misconduct by anyone working on the study. Depending on the infraction, consequences can range from a publicly posted warning letter by the FDA to criminal prosecution and fines or imprisonment.

**Conclusion:** Investigators are not only responsible for producing high-quality, meaningful, scientific research, but they are also responsible for maintaining public trust. If the principal investigator acts with integrity and provides training and oversight of employees, FDA inquiries should go smoothly. Following good clinical practice standards for clinical research will result in quality data collection and facilitate the analysis and publication process.

## INTRODUCTION

More than 300,000 clinical trials were listed on clinicaltrials.gov as of July 2019, of which 79% were interventional, and more than 20,000 of these trials were actively recruiting in the United States.^1^ Well-designed and properly executed clinical trials are integral to the development of new drug, device, surgical, and behavioral interventions. Healthcare facilities that conduct clinical trials can provide novel, otherwise unavailable treatments for their patients. The individual responsible for the conduct of a clinical study at a site is the principal investigator (PI). While there are many advantages to being a PI for a clinical trial, PIs are subject to numerous restrictions designed to ensure patient safety and meaningful study results. A number of regulatory documents govern investigator conduct in clinical trials in the United States, including Title 21 of the Code of Federal Regulations, the Federal Food, Drug, and Cosmetic Act (21 CFR)^2^; the International Conference on Harmonisation (ICH) Guideline for Good Clinical Practice (GCP)^3^; and the US Food and Drug Administration (FDA) Form 1572.^4^ The ICH was formed to bring together regulatory authorities and pharmaceutical companies from around the world to ensure that safe, effective, and quality-controlled medications are developed and registered efficiently. While the ICH guidelines are not law, GCP is a standard that researchers in the United States and abroad recognize and abide by. The CFR and FDA Form 1572 are legally binding in the United States. Despite these regulations and guidelines, the FDA issues hundreds of warning letters each year for noncompliance.^5^

This article examines the contents of the above-mentioned documents and strategies for operating within regulatory guidelines for FDA-regulated research on investigational drugs and devices. The goal of this article is to demystify the regulations regarding PI responsibilities in clinical trials.

## PRINCIPAL INVESTIGATOR OBLIGATIONS

A PI need not be a physician. Sponsors who design and pay for clinical trials (eg, pharmaceutical companies) are required by law^6,7^ to select individuals who are qualified by training and experience to conduct a clinical trial. However, if the PI is not a physician, a qualified physician or dentist should be listed as a subinvestigator for the trial and assume responsibility for all trial-related medical or dental decisions.^8^

A PI can outsource research tasks, such as analysis of blood biomarkers, to contract research organizations (businesses with specialized expertise in services that are too expensive or time-consuming to conduct in-house). However, the PI—not the contract research organization—is responsible and accountable for the research.

The PI's responsibility also includes the research staff. Staff members may perform study-related tasks, such as consenting a subject, but the FDA stipulates, “The investigator should ensure that any individual to whom a task is delegated is qualified by education, training, and experience (and state licensure where relevant) to perform the delegated task.”^9^ The PI is held accountable if anything goes wrong. Investigators are responsible for any breach of protocol, discrepancies in recordkeeping, or other infractions by subordinates. The concept of complete responsibility being vested in the PI is also present in the ICH international guidelines.^3^ Tasks and day-to-day management of the trial may be delegated, but the investigator is responsible for ensuring that the trial is conducted compliantly and that recordkeeping is appropriate and up to date. PI responsibilities are summarized in the Table.

**Table.** Summary of Investigator Requirements and Responsibilities

Category	Requirements and Responsibilities
Principal investigator	•Anyone qualified by training to run the trial; a physician or dentist must be listed as a subinvestigator if the principal investigator is not a physician
	•Hire and train qualified individuals to run the trial
Subject safety	•Protect subjects from harm
	•Keep track of drugs and distribute only as specified in the protocol
	•Obtain informed consent
	•Ensure IRB approval
	•Keep careful records and maintain them for as long as the protocol dictates or at least 2 years
Reports	•Progress, safety, financial, and a final report to the study sponsor
	•Adverse events; serious adverse events must be reported immediately
	•Update financial disclosures if any circumstances change during the study
Form 1572	•Strictly adhere to the protocol
	•Directly supervise the study and take responsibility for study staff
	•Inform subjects of experimental nature of the drug products
	•Report adverse events and stay updated on the investigational brochure
	•Maintain records
	•Ensure IRB compliance
FDA inspections	•Ensure all records are complete and easily accessible by FDA
	•Send a written response within 15 business days if any violations are found
How to avoid violations	•Read all communications from the IRB
	•Hire experienced staff and verify their credentials
	•Train staff regularly
	•Check for conflicts of interest/financial disclosures regularly
	•Write efficient protocols or reduce inefficiencies or confusing portions of the protocol
	•Keep regulatory binders up to date and conduct continuing reviews
	•Meet with the team regularly
	•Conduct several dry runs to ensure the study will run smoothly
	•Regularly check data processes

FDA, US Food and Drug Administration; IRB, institutional review board.

### Protecting Subjects

The 1962 Kefauver-Harris Amendments increased FDA regulation of clinical trials after thousands of pregnant women in Europe, Canada, and other countries were prescribed thalidomide for nausea, resulting in widespread birth defects in their children.^10^ This important tragedy served as an incentive for stringent toxicology testing and oversight of investigational new drugs.^11^

Investigators are charged with protecting the rights, safety, and welfare of subjects; controlling drug storage and distribution; ensuring that informed consent is adequately obtained according to 21 CFR §50;^12^ and ensuring that institutional review board (IRB) review, approval, and reporting requirements are met per 21 CFR §56.^13^ Recordkeeping and retention requirements include maintaining adequate records of the disposition of the drug, histories that record all observations, and any other data relevant to the individual trial.^14^ These records must be maintained for 2 years following the marketing application approval for the drug indication that was studied or 2 years after the FDA is notified that no application will be filed for the indication.

### Reporting

Investigators must send progress reports, safety reports, and financial disclosures to the sponsor at least annually during the trial, and a final report is due at the end of the trial as specified in 21 CFR §312.64.^15^ Serious adverse events—unexpected events that are severe or life-threatening—must be reported immediately, but nonserious adverse events can be reported according to the timetable for reporting specified in the protocol. The required reports for medical devices are outlined in 21 CFR §812.150.^16^ Financial disclosures to the sponsor should be updated as necessary during the investigation and for 1 year following study completion.^17^ Having a significant equity interest in the sponsor, which is any stock in a non–publicly traded company or $50,000 of stock in a publicly traded company, could represent a conflict of interest for the investigator, subinvestigator, or study staff. Even if the stock is held by spouses and/or dependent children of those conducting the trial, the financial interest must be disclosed to the study sponsor. Financial disclosures should be promptly updated if they change during the study.

### Completing US Food and Drug Administration Form 1572

Statement of Investigator, FDA Form 1572, is used when a sponsor and investigator initiate a new trial conducted under an investigational new drug application. Form 1572 provides the study sponsor with information about the investigator's qualifications and site-specific details. This information enables the sponsor to document that both the site and investigator are appropriate for a clinical investigation. The form includes a confirmation that the investigator acknowledges and is committed to follow pertinent FDA regulations during the study.

The sponsor must ensure that Form 1572 is completed prior to permitting an investigator to begin a study,^6^ and the PI should not sign the form until he or she has a full understanding of the protocol, the potential risks as outlined in the investigational brochure (a document provided by the sponsor that details all prior experience with the investigational drug or device), and the commitments listed on the form (Figure).

**Figure.**
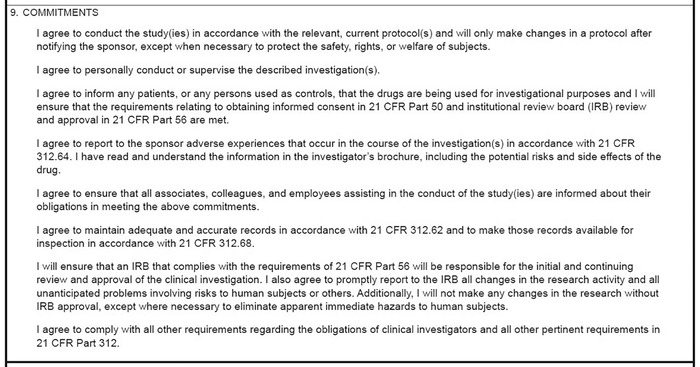
**Investigator commitments from US Food and Drug Administration Form 1572.**

By signing Form 1572, the PI guarantees to personally conduct or directly supervise the study. This guarantee requires that all study personnel be informed of their obligation to strictly adhere to the protocol unless the sponsor is notified or a subject is at risk of health complications. If adverse events occur, they must be reported to the sponsor.

As stated earlier, while study staff may run day-to-day operations, ultimately, the investigator is responsible for overseeing and enforcing compliance with the protocol and ensuring that informed consent has been obtained from all subjects.^12^ Additionally, appropriate IRB review, approval, and reporting must be completed according to the CFR.^13^ If any unanticipated problems occur that could cause risks to a subject, they must be reported to the IRB as soon as possible. Subject safety is the priority, and reporting should occur after any immediate threats to a subject are addressed. If these problems indicate a need for changes to the protocol, the PI is required to submit the changes to the IRB for approval.

Form 1572 is not required for medical device clinical trials, but 21 CFR §812.43^7^ stipulates that for these studies, PIs sign an agreement that includes the investigator's curriculum vitae; a statement of relevant experience; and a commitment to follow the protocol and all regulations, to supervise all testing of the device involving human subjects, and to ensure that informed consent is obtained, properly documented, and retained. If the PI was involved in a study that was terminated, the investigator must explain why. Finally, financial disclosures must be submitted for medical device trials and updated throughout the study if they change.^18^

## FOOD AND DRUG ADMINISTRATION INSPECTIONS

The FDA can conduct both announced and unannounced inspections to verify compliance with the CFR and that data submitted to the agency are accurate. Inspections may be triggered by sponsor concerns, a complaint about the conduct of a site, termination of a clinical site, or the request of an FDA review division. Additionally, the FDA may inspect a site during an ongoing clinical trial to spot check in real time that the site is running compliantly and may conduct inspections for special interest products. For example, if many sites are investigating a new drug that has caused problems such as injuries or deaths at other sites, the FDA may inspect other sites trialing the same drug. The CFR gives the FDA the authority to access, copy, and verify clinical study records.^19,20^

Risk factors for noncompliance include poor supervision and training of study staff, lack of investigator involvement, delegation of study tasks to unqualified persons, failure to adequately protect study subjects, and an overworked PI and study staff. To determine if PI supervision is adequate, the FDA assesses (1) if the study staff are qualified to perform delegated tasks, (2) if the study staff received adequate training and explanation of the protocol, (3) if the PI's involvement is adequate and ongoing, and (4) if involved third parties and contract research organizations are adequately supervised. Thus, it is crucially important that the PI is regularly involved in the study and hires competent and qualified individuals to work on the study. By appropriately training and assigning study staff and remaining involved in the study, PIs can significantly reduce the risk of noncompliance.

## VIOLATIONS

If the FDA inspectors find violations made by any study staff, a warning letter will be issued to the PI and publicly displayed on the FDA website to ensure that sponsors do not unknowingly engage with offenders.^5^

FDA Form 483^21^ is used to notify senior management of violations at the study site. After the inspection, management and FDA inspectors will discuss each infraction to ensure that both parties understand the citation. The investigator should send a written response to the FDA within 15 business days addressing each concern. If the infractions are severe and/or not addressed appropriately or not addressed at all, the FDA can issue a Notice of Initiation of Disqualification Proceedings and Opportunity to Explain, disqualify an investigator, pursue criminal investigation through the Office of Criminal Investigations, or pursue debarment. Disqualification procedures are outlined in 21 CFR §312.70^22^ and 21 CFR §812.119^23^ for repeated and deliberate noncompliance. If repeated and deliberate noncompliance is noted, the FDA will give the investigator the opportunity to explain citations informally, as well as the opportunity for a formal hearing. If the FDA rules against the investigator, the individual may be temporarily or permanently banned from participating in trials of investigational drugs or medical devices. Debarments are listed on the FDA website.^24^

The Ketek case provides multiple examples of egregious clinical trial violations and problems at the FDA. Ketek (telithromycin), developed by the pharmaceutical company Sanofi-Aventis, was the first antimicrobial to circumvent macrolide resistance and was approved by the FDA in 2004 for community-acquired respiratory tract infections. After approval, however, the drug was linked to severe liver injury in dozens of patients, including 4 fatalities, prompting urgent safety warnings.^25^ Two congressional investigations were conducted into the FDA actions surrounding the drug approval.

The integrity of “Study 3014” was questionable. Many of the investigators were inexperienced, and Sanofi-Aventis was paying them $400 per enrolled patient. The top-enrolling physician had more than 400 patients enrolled and was found to have entirely fabricated numerous study participants. She was prosecuted and imprisoned, and 4 other sites were criminally investigated. Despite these problems, FDA employees who were not involved in the investigation presented the drug for approval without mentioning the massive breaches of data integrity. An article by Dr David Ross outlines the timeline and details of how Ketek was approved for the market,^26^ but the bottom line is that inexperienced or corrupt investigators, falsified data, and irresponsible actions by FDA managers led to inappropriate drug approval and patient deaths. In 2007, the FDA dropped two of the drug indications, limiting the antibiotic to use for community-acquired pneumonia, and myriad boxed warnings accompanied the labeling. In 2016, Sanofi-Aventis permanently discontinued the drug.

## MAINTAINING COMPLIANCE

PIs who are involved in their studies and proactively improve processes are unlikely to have disciplinary action result from FDA investigations. Guidelines for PIs for maintaining compliance are as follows:

1. Read all communications from the IRB and ensure that the study team is compliant with IRB requests and determinations.
2. Ensure study staff are experienced and verify their credentials, but do not assume they have been adequately trained by a previous employer.
3. Institute regular training and assessments for subject visits, laboratory testing, data input and handling, drug accountability, and recordkeeping.
4. Ensure that the study staff have disclosed any conflicts of interest and understand the importance of doing so.
5. Streamline the protocol, ensuring that it is straightforward with standardized data input, checklists, and minimal amendments.
6. Keep regulatory binders up to date with current training certifications and licensure. Ensure that continuing reviews are done annually and submitted to the IRB.
7. Hold weekly team meetings and make plans for disasters, emergencies, quality assurance, data safety, and data management.
8. Institute study dry runs before patients are involved to ensure that no components of consenting a patient or documenting a visit are missing.
9. Implement regular data input, data cleaning, and query answering to keep systemic problems from spiraling out of control. If changes are made to data, audit the trail of changes to document why data were changed and by whom.
10. Determine if infractions require individual correction or if systemic changes need to be implemented to prevent similar problems from arising in the future.

Although developing standard operating procedures and conducting regular self-assessments may seem to be a great deal of superfluous work, these activities will decrease discrepancies in data collection and will ensure timely reporting of any issues.

## CONCLUSION

Investigators are not only responsible for producing high-quality, meaningful, scientific research, but they are also responsible for maintaining public trust. High-profile infractions such as the Ketek study cause public distrust and patient fatalities, but even small infractions such as waiting a few weeks to input study data can lead to patient safety issues. Newcomers to clinical trials should work with an experienced mentor who can help structure standard operating procedures for trials and employee training. If a PI acts with integrity and provides training and oversight of employees, FDA inquiries should go smoothly. Regardless of governmental oversight, using GCP standards for clinical research will result in quality data collection and facilitate the analysis and publication process.

## Notes

This article meets the Accreditation Council for Graduate Medical Education and the American Board of Medical Specialties Maintenance of Certification competencies for Patient Care, Medical Knowledge, and Systems-Based Practice.
